# Major ocean currents may shape the microbiome of the topshell *Phorcus sauciatus* in the NE Atlantic Ocean

**DOI:** 10.1038/s41598-021-91448-0

**Published:** 2021-06-14

**Authors:** Ricardo Sousa, Joana Vasconcelos, Iván Vera-Escalona, João Delgado, Mafalda Freitas, José A. González, Rodrigo Riera

**Affiliations:** 1 Observatório Oceânico da Madeira, Agência Regional para o Desenvolvimento da Investigação Tecnologia e Inovação (OOM/ARDITI) – Edifício Madeira Tecnopolo, Funchal, Madeira, Portugal; 2 Direção Regional do Mar, Direção de Serviços de Monitorização, Estudos e Investigação do Mar (DRM/DSEIMar), Lota do Funchal 1º piso, Rua Virgílio Teixeira, 9004-562 Funchal, Madeira, Portugal; 3 MARE - Marine and Environmental Sciences Centre, Agência Regional Para o Desenvolvimento da Investigação Tecnologia e Inovação (ARDITI), Edifício Madeira Tecnopolo Piso 0, Caminho da Penteada, 9020-105 Funchal, Madeira, Portugal; 4 Faculdade de Ciências de Vida, Universidade da Madeira, Campus Universitário da Madeira, Caminho da Penteada, 9020-020 Funchal, Madeira, Portugal; 5 Departamento de Ecología, Facultad de Ciencias, Universidad Católica de la Santísima Concepción, Casilla 297, Concepción, Chile; 6 Centro Interdisciplinar de Investigação Marinha e Ambiental (CIIMAR/CIMAR), Porto, Portugal; 7 Ecología Marina Aplicada y Pesquerías (I-UNAT), Universidad de Las Palmas de Gran Canaria, Las Palmas de Gran Canaria, Spain; 8 IU-ECOAQUA, Group of Biodiversity and Conservation (BIOCON), Universidad de Las Palmas de Gran Canaria, Las Palmas de Gran Canaria, Spain

**Keywords:** Molecular biology, Ecology

## Abstract

Studies on microbial communities are pivotal to understand the role and the evolutionary paths of the host and their associated microorganisms in the ecosystems. Meta-genomics techniques have proven to be one of the most effective tools in the identification of endosymbiotic communities of host species. The microbiome of the highly exploited topshell *Phorcus sauciatus* was characterized in the Northeastern Atlantic (Portugal, Madeira, Selvagens, Canaries and Azores). Alpha diversity analysis based on observed OTUs showed significant differences among regions. The Principal Coordinates Analysis of beta-diversity based on presence/absence showed three well differentiated groups, one from Azores, a second from Madeira and the third one for mainland Portugal, Selvagens and the Canaries. The microbiome results may be mainly explained by large-scale oceanographic processes of the study region, i.e., the North Atlantic Subtropical Gyre, and specifically by the Canary Current. Our results suggest the feasibility of microbiome as a model study to unravel biogeographic and evolutionary processes in marine species with high dispersive potential.

## Introduction

During the last decades we have observed an increase in the number of studies trying to elucidate the role of species and the environment where they live due to research expeditions and the use of several modern techniques to identify species, including genetic-based techniques^1^. Early studies based on genetics focused on the description of species and populations but soon after the first results, it was evident that genetic-based studies could also be used to identify and describe major biogeographic patterns as well as to create the pathway to evaluate new hypotheses and ecological questions^2–4^.

Genetic techniques have become more affordable which has allowed to explore into more species and more environments^5,6^. Among this new genetic-based techniques, are those based on high-throughput sequencing methods that have provided a great amount of information regarding the diversity of organisms, including microbial communities^7–10^. At microbial level, this new technique enables characterization of community composition and dynamics, including rare phylotypes^11^ that can be used as seed banks^12^. Genetic-based methods have shown that marine species can be more diverse and with singular patterns than expected, but also that marine host-associated microbiome has been shown to be highly diverse^13^. Microbiome studies have been focused mostly on a restricted number of marine organisms, including sponges, cnidarians or echinoderms^14–17^, and chordates such as, sea squirts^18^ and sharks^19^. Microbiome studies on other groups remain poorly studied and when existing, they have been mostly focused on commercial-interest species such as, molluscs, e.g. abalones^20^.

It is necessary to increase our knowledge in organisms besides those mentioned earlier, especially since it is known that marine environments host a vast diversity of microorganisms that constitute the main support of the remaining life in the ocean^21^. The study of microbiome has an enormous potential to be used as a proxy to disentangle ecological and evolutionary processes such as, patterns underlying community assembly, life history or biogeographic patterns, among others. In this context, microbiome may shed light from small-scale (e.g., site-specificity) to large-scale patterns such as, major ocean currents. For instance, Dick et al.^22^ observed that microbiome associated to hydrothermal vents is shaped by geographic isolation and limited dispersal of these deep-sea and sparse communities. Although biogeographic patterns are usually described for conspicuous, they can also be described using the microbiome living within organisms, but this aspect remains scarcely studied. This is especially true for the microbiome of organisms from highly isolated areas (e.g., oceanic islands and archipelagos), where biogeographic patterns are less studied.

Most of the information concerning the influence of oceanographic processes such as major currents are based on pole-to-pole^23^ and shallow-to-deep water^24^ studies on the distribution of microorganisms in the marine realm. However, the microbiome from marine species have been neglected in the above-mentioned studies, including taxa with planktonic dispersal stage in which the highly connectivity nature of the pelagic realm greatly determines their distribution^25^. For instance, if ocean currents can explain the general distribution of marine coastal species, it should be likely to expect that currents may also explain the microbiome biodiversity of their host marine species. Surprisingly, this hypothesis has remained mostly overlooked in studies focused on marine microbiome.

With this in mind, we herein used individuals of the topshell *Phorcus sauciatus* (Koch, 1845) as a case study to describe their microbiomes and understand how major currents can affect the biodiversity of their microbiome along the Macaronesian islands. *Phorcus sauciatus* is a microphagus herbivore with a widespread distribution in the NE Atlantic Ocean, from the Iberian Peninsula to North Africa mainland and the Macaronesian archipelagos^26–29^. We herein isolated and identified endosymbiotic bacterial strains from the digestive system and gonads of the topshell *P. sauciatus* from mainland Portugal, and from several inhabited Macaronesian archipelagos, namely Azores, Madeira and Canary Islands, and one uninhabited Macaronesian archipelago, Selvagens Islands, considered a pristine spot due to the lack of human presence. We expect *P. sauciatus* microbiome to show clustering patterns depending on the area, since connectivity of Macaronesian archipelagos is low among them and with mainland Portugal. Specifically, we herein aim (i) to identify host-specific microbiome of an intertidal exploited species, the mollusc *P. sauciatus*; (ii) to evaluate the variations in host-associated microbiome across a latitudinal gradient in isolated archipelagos and adjacent mainland; (iii) to discern the importance of small-scale processes, i.e., environmental selection, and large-scale processes, i.e., geographic distances among populations in shaping microbiome of *P. sauciatus*; and (iii) to explore the potential of microbiome to unravel biogeographic differences among locations and the importance of large-scale oceanographic processes, i.e., major currents, structuring microbiome composition of this species.

## Materials and methods

### Sample collection, DNA extraction and polymerase chain reaction amplification of 16S rRNA genes

Individuals of the mollusc *P. sauciatus* were collected by hand from 5 intertidal locations (n = 50; Fig. 1) including mainland Portugal (n = 10) and the islands of Madeira (n = 10), Selvagens (n = 10), Azores (n = 10) and Canaries (n = 10) to identify the endosymbiotic bacterial species contained in the digestive system and gonads. DNA was extracted with an E.Z.N.A. Mollusc DNA kit using the manufacturer protocol. Genomic DNA quality was determined in a 1.5% agarose gel electrophoresis and quantified by fluorometry. Extracted DNA was then forwarded for 16S target PCR amplification, library construction and sequencing using 16S Metagnomic Sequencing Library preparation and protocol and the MiSeq Reagent Kit v3 in an ILLUMINA MiSeq Platform. Regions amplified corresponded to the V3 and V4 regions of the 16S rRNA gene using the primers Pro341F (5'-CCTACGGGNBGCASCAG')^30^ and Bact805R (3'-GACTACHVGGGTATCTAATCC')^31^. All extractions, amplifications and sequencing analyses were made by STABVIDA, LDA.

**Figure﻿ 1:**
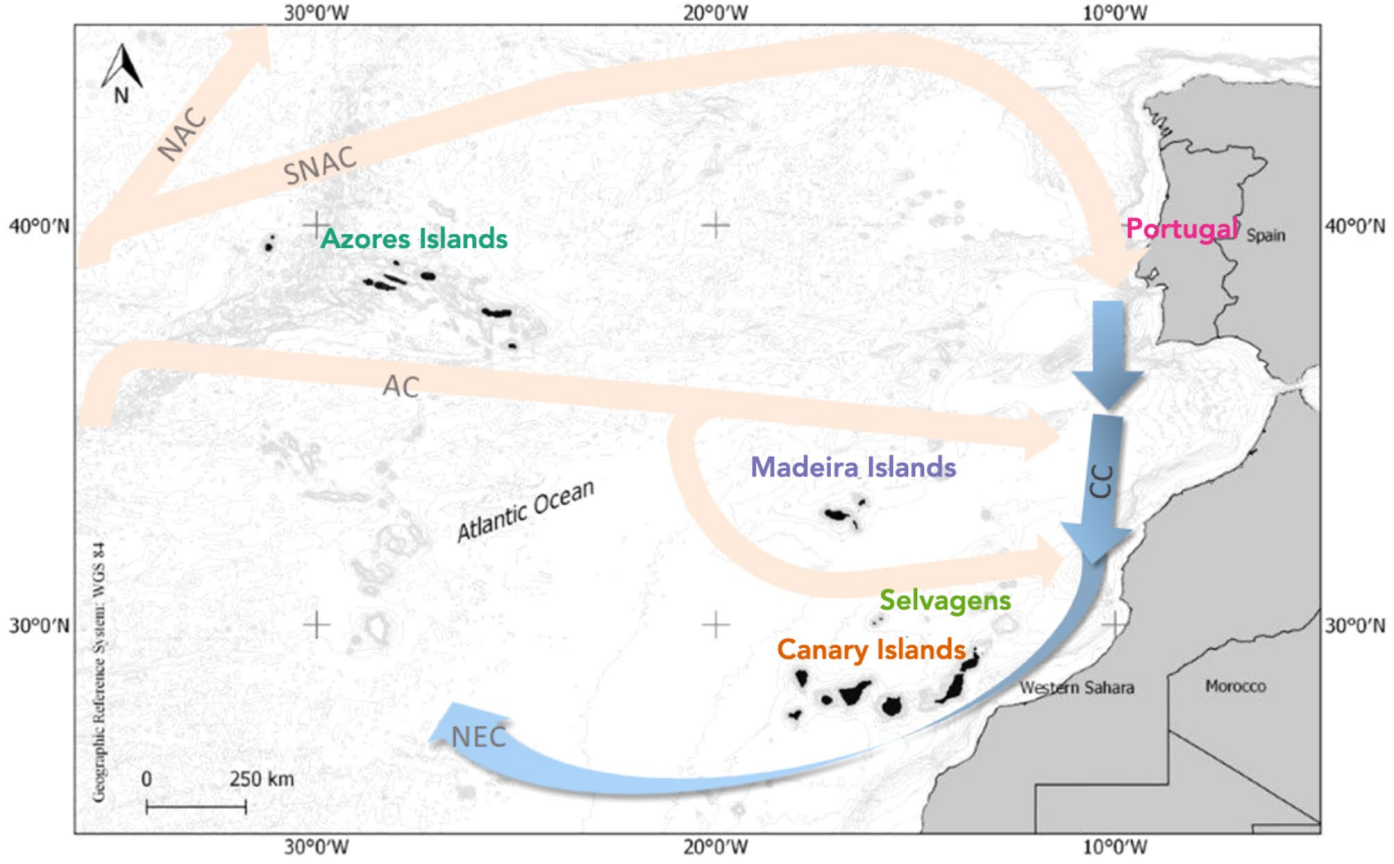
Sampling locations of the mollusc *Phorcus sauciatus* in coastal areas from the Atlantic Ocean (mainland Portugal and the islands of Madeira, Selvagens, Azores and the Canaries) including the North Atlantic Current (NAC), Southern limb of the North Atlantic Current (SNAC), Azores Current (AZ), Canary Current (CC), and the North Equatorial Current (NEC). Map made with QGIS (v 3.6, https://www.qgis.org)^32^.

### ILLUMINA high-throughput 16S rRNA gene sequencing and bioinformatics analyses

QIIME2 v2018.6 pipeline was used for data processing of the endosymbiotic composition of the 50 samples of *P. sauciatus* sequenced. Paired-end sequence reads ranged from 124,304 to 1,743,120 within location with samples from Madeira and Selvagens generating the lowest number of sequences reads. Collected reads were deposited in Genbank (Access Code 602319) under the Projects MARISCOMAC (MAC/2.3d/097) and MACAROFOOD (MAC/2.3d/015). Forward and reverse reads were merged using SeqPrep and classified to their respective samples according to their barcodes. Sequences were then screened by quality and size, and de-replicated. The resulting file was checked for chimeric sequences with SILVA_123 database using UCHIME^33^.

### Taxonomic identification within and among groups

Sequences were classified by taxon using the SILVA release 132 QIIME database, with a clustering threshold of 97%. OTUs with less than 0.05% of abundance, and OTUs classified as chloroplast or mitochondria were removed from the dataset. OTU table was rarefied to 1154 reads based on the lowest number of the post-assembly and filtered sequences in a sample for comparisons across samples^34^. Interactive plots of the taxonomic profiles were visualized as plot bars with taxonomic identification at different levels, from Bacteria groups to genus. The identification at genus level depended on the sequence length and the number of previously identified species in Genbank. Therefore, not all OTUs were identified at species level.

### Alpha diversity metrics

**Figure 2:**
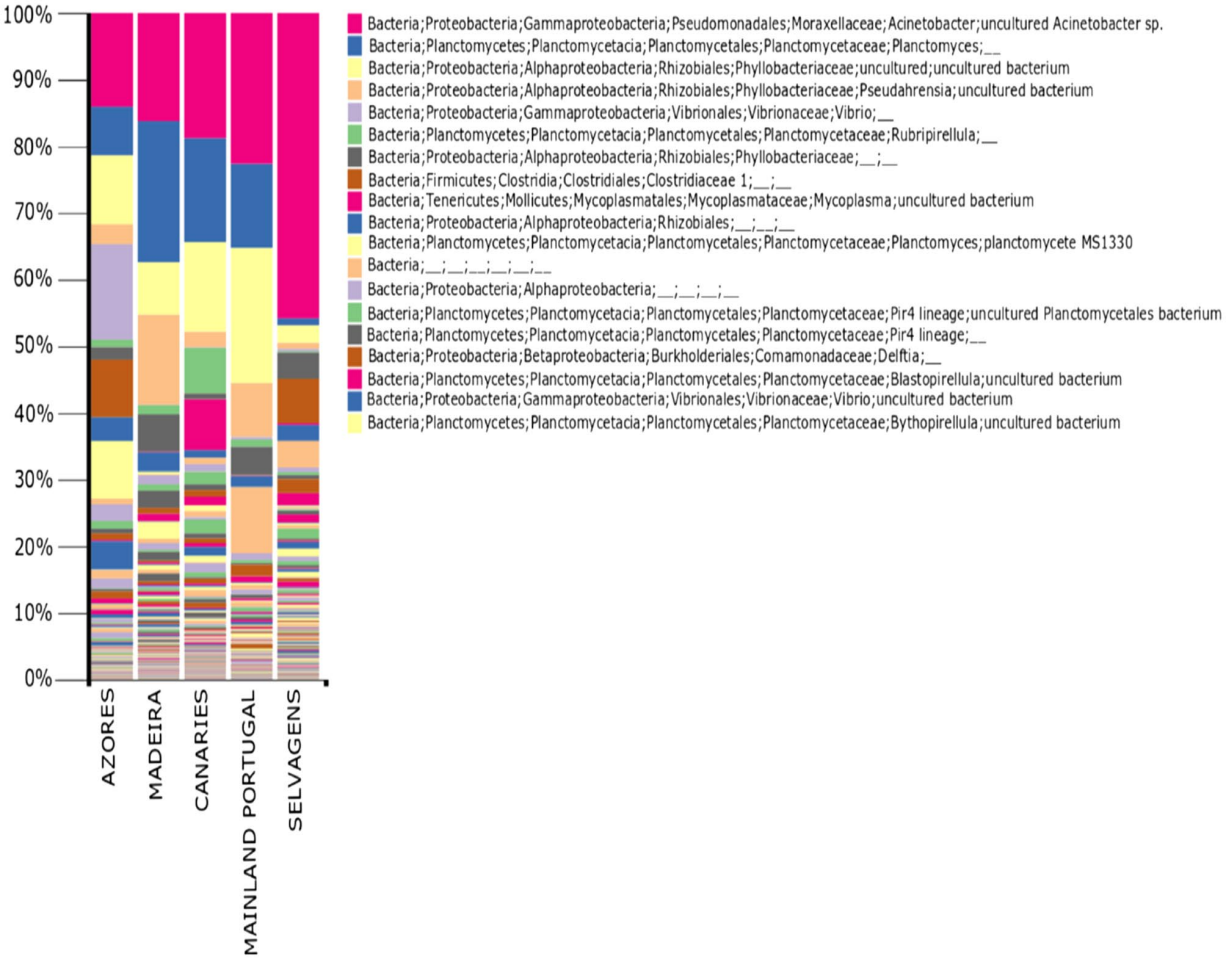
Operation Taxonomic Units (OTUs) at species level for endosymbiotic bacteria found in the mollusc *Phorcus sauciatus* from five coastal locations in the Atlantic Ocean (mainland Portugal and the islands of Madeira, Selvagens, Azores and the Canaries).

Alpha rarefaction curve was used to determine the confidence of the taxonomic species identification and comparisons between groups. This parameter depends on the quality and the amount of the DNA extraction. A plateau on the rarefaction curve of a sample suggests that the sequencing was able to capture most taxa. On the opposite, a steep curve suggests that not all taxa were captured in the analysis (Fig. 2). Hence, the analysis allows to identify the presence of sequences with low sequencing depth and removed them from further analyses. A summarized table of features and samples was used to determine the minimum number of samples before calculating all following alpha and beta diversity analyses. Alpha diversity was calculated as species richness based on the number of OTUs in a sample as well as with Shannon and Simpson estimate species diversity for each rarified table quantifying bacterial diversity within each sample-based similarity and similarity weighted by dominant species respectively. Mean values were used for statistical test and plots using Kruskal–Wallis tests to estimate differences in alpha diversity among groups.

### Beta diversity

Beta-diversity based analyses were used to measure the degree of differentiation between the samples, revealing microbial aspects that are not observable from the composition of individual samples. Beta-diversity statistics were calculated using the Jaccard distances and Bray–Curtis dissimilarity based on presence/absence of sequences and abundance respectively. Along with these Beta diversity statistics, Unifrac unweighted and weighted analyses were used to consider the effect of sequences purely based on sequences distances and branch lengths weighted by relative abundances. Distance matrices, obtained from a rooted phylogenetic tree built with QIIME, were then plotted using a Principal Coordinates Analysis (PCoA) to visualize similarities or dissimilarities among the 50 samples. The statistical significance of the clusters among samples were calculated with a permutational multivariate analysis of variance (PERMANOVA) with sample size = 50, number of groups = 5 and 999 permutations.

## Results

### Alpha rarefaction

The alpha rarefaction analysis revealed that, in general, most of samples had a high sequencing depth with a cutoff value of 1154 reads depth in one of the samples from Selvagens. Therefore, all following analyses were rarefied to 1154 reads frequency.

### Taxonomic identification

The identification at Level 7 was able to recover organisms at species level and other higher taxonomic ranks (Fig. 2). A total of 910 OTUs were identified, with *Acinetobacter* being the most frequent group identified followed by *Planctomyces* and unidentified members of the family Phyllobacteriaceae. In terms of the most abundant families, Portugal, Madeira, and Canaries were more similar and Azores and Selvagens the less similar among the five sampled populations. Although individuals from Azores showed a high abundance of *Acinetobacter*, *Planctomyces*, and unidentified members of the family Phyllobacteriaceae, the most abundant OTUs belonged to the genus *Vibrio*. The most abundant OTUs in Selvagens belonged to the genus *Acitenobacter*, followed by Clostridiaceae and unidentified Bacteria. In this population, the abundance of *Acitenobacter* was sevenfold higher than the following identified OTUs, a very unusual abundance difference when compared with the observed abundance in the remaining four populations.

### Alpha diversity

**Figure 3:**
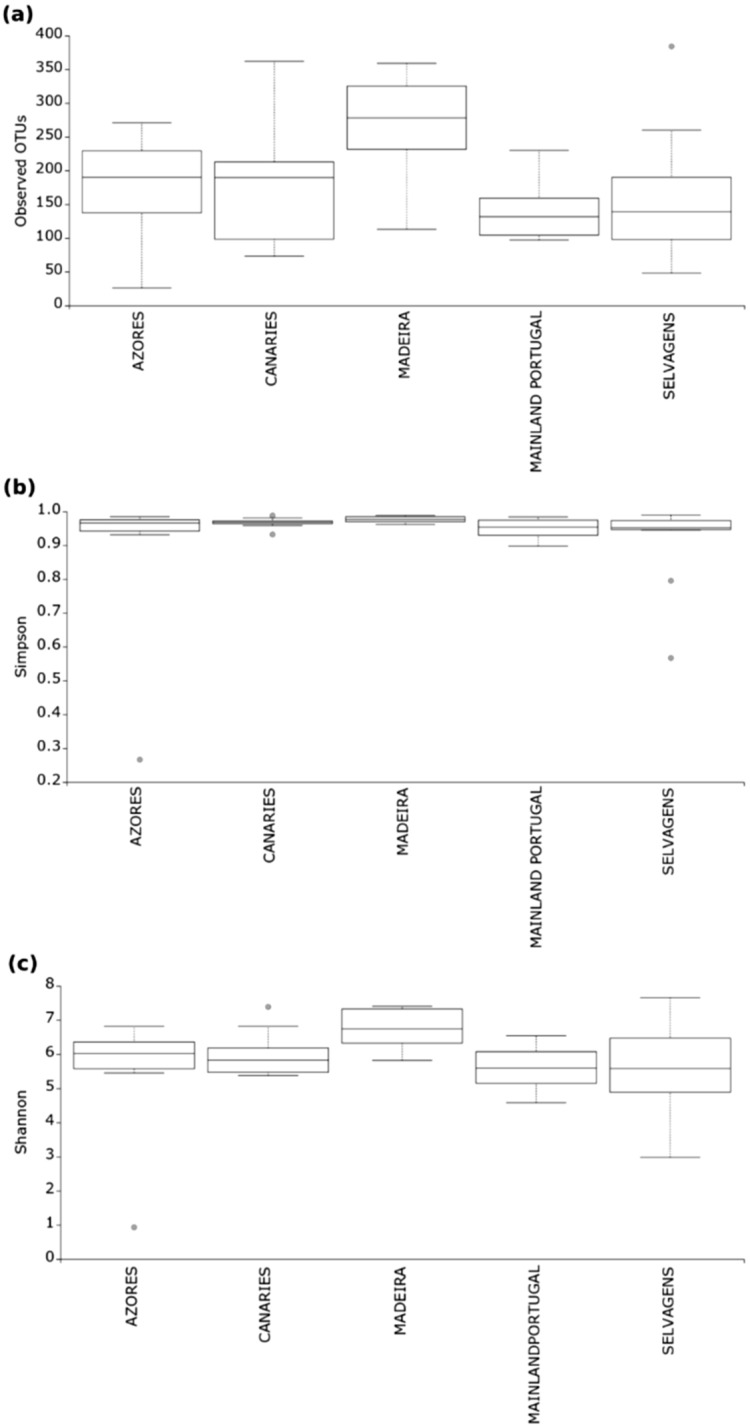
Alpha diversity indices based on Number of OTUs: (**a**) Simpson similarity (**b**) and Shannon function (**c**) for 5 intertidal populations (with 10 replicates each) of the microbiome of the mollusc *Phorcus sauciatus* in the Atlantic Ocean (mainland Portugal and the islands of Madeira, Selvagens, Azores and the Canaries).

This diversity of observed OTUs greatly varied among all groups (H = 12.501, *p* = 0.014), mainly explained by the significant differences between Madeira and the remaining sampling regions (Azores-Madeira, H = 6.222, *p* = 0.012; Madeira-Selvagens, H = 5.855, *p* = 0.016; Madeira-Canaries, H = 4.480, *p* = 0.034; Madeira-mainland Portugal, H = 10.087, *p* = 0.001) (Fig. 3a). However, Simpson diversity revealed non-significant differences when comparing all groups (H = 7.824, *p* = 0.099), but significant differences between Madeira-mainland Portugal (H = 5.143, *p* = 0.023) and differences at the edge of significance between Madeira and Selvagens (H = 3.57, *p* = 0.058) (Fig. 3b). Shannon diversity showed significant differences among groups (H = 1.513, *p* = 0.021) explained by the differences between Madeira and the remaining sampling regions (Madeira-Azores, H = 5.491, *p* = 0.019; Madeira-Selvagens, H = 4.805, *p* = 0.028; Madeira-Canaries, H = 5.851, *p* = 0.016; Madeira-mainland Portugal, H = 8.691, *p* = 0.003) (Fig. 3c).

### Beta diversity

**Figure 4:**
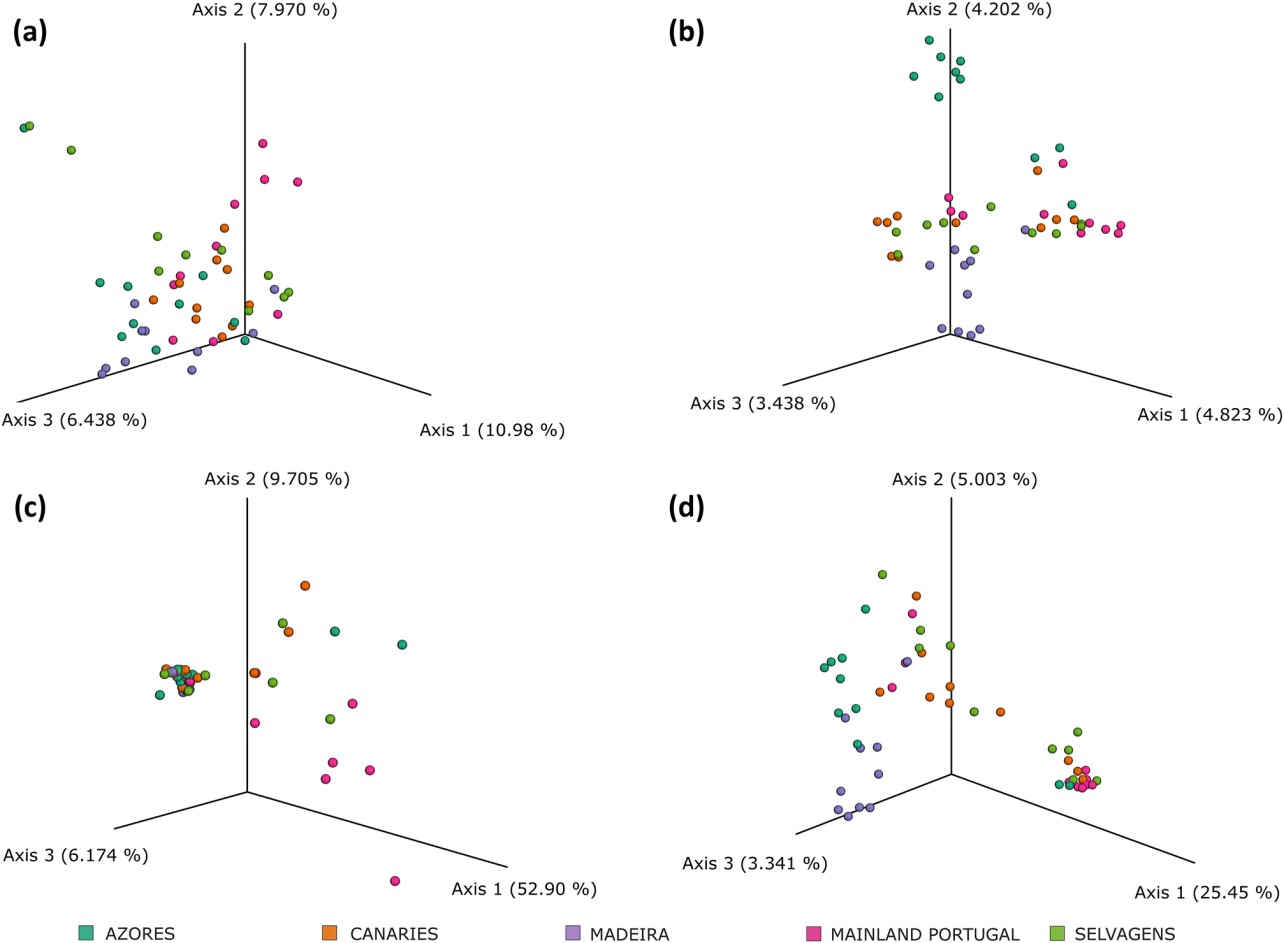
Principal Coordinates Analyses of the Beta diversity using the Bray Curtis (**a**), Jaccard Index (**b**), Weighted Unifrac (**c**), and Uniweighted Unifrac (**d**) for 50 samples of the microbiome of the mollusc *Phorcus sauciatus* from 5 intertidal locations in the Atlantic Ocean (mainland Portugal and the islands of Madeira, Selvagens, Azores and the Canaries).

The Principal Coordinates Analysis (PCoA) of beta-diversity distances based on Bray–Curtis dissimilarities (absence/presence and abundance of species) showed that only individuals from mainland Portugal were slightly differentiated (Fig. 4a, *p* = 0.001). PCoA analysis created from distances based on the Jaccard Index (absence/presence of species) showed three well-differentiated groups (Fig. 4b, *p* = 0.001). One group formed by individuals from Azores and a second group formed by individuals from Madeira. A third group included all samples from mainland Portugal, Selvagens, Canaries and one sample from Azores. A similar trend is observed in the PCoA based on the Unifrac unweighted analysis (absence/presence of species) (Fig. 4d) but not in the PCoA based on Unifrac weighted (absence/presence and abundance of species; Fig. 4c).

**Figure 5:**
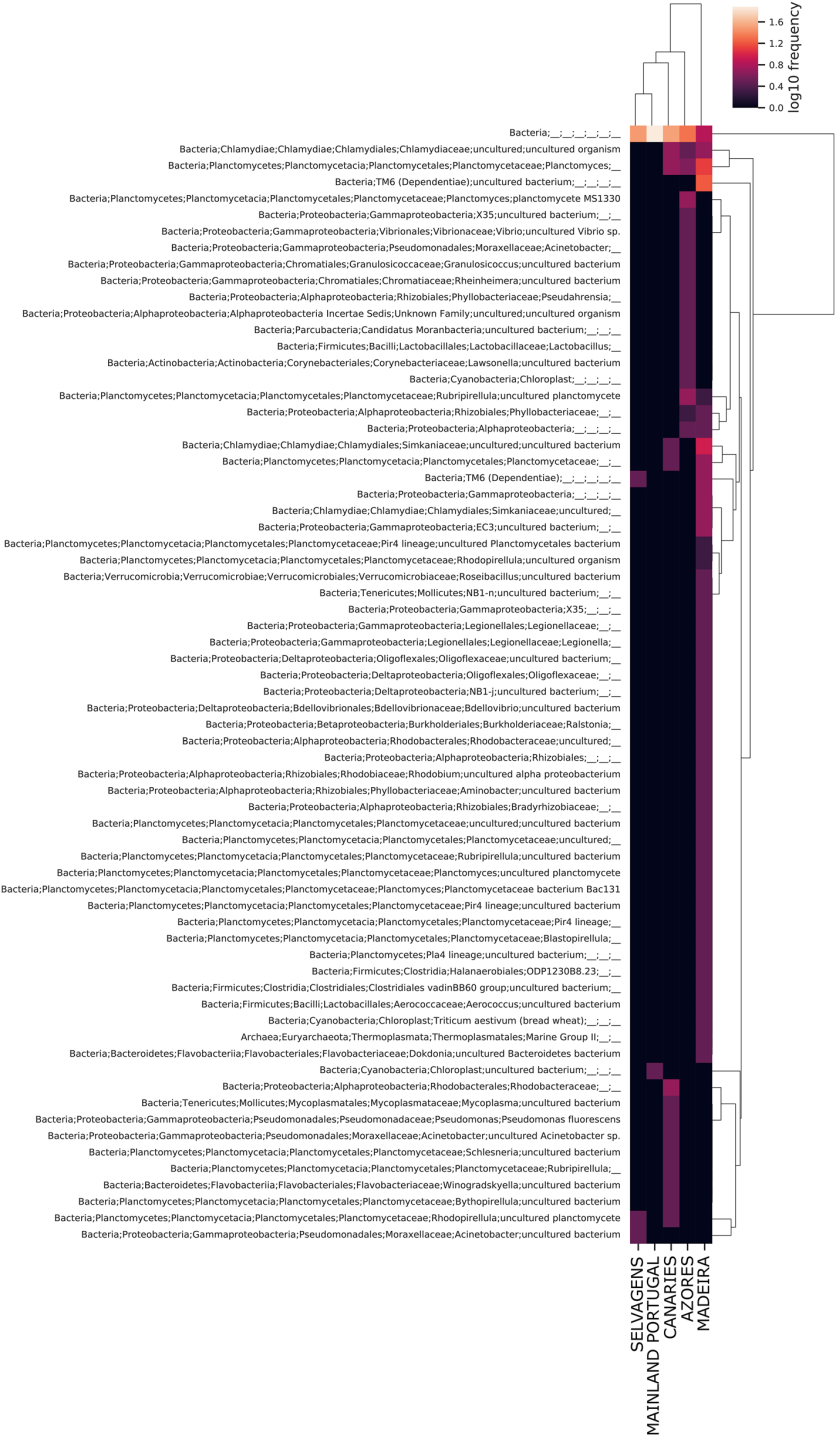
Heatmap with less abundant species (frequency 0–2) in the microbiome of the mollusc *Phorcus sauciatus* from five coastal locations in the Atlantic Ocean (mainland Portugal and the islands of Madeira, Selvagens, Azores and the Canaries).

The heatmap (Fig. 5) showing only species with low frequency suggested that the presence of these species may explain the observed pattern in the PCoA based on the Jaccard distances.

PERMANOVA (Table 1) analysis based on Bray–Curtis and Jaccard indices revealed that the microbiome within *P. sauciatus* was highly differentiated along the five sampled locations, a different trend than the observed with UniFrac analyses where by considering sequence distances it is observed that Canaries and Selvagens were less differentiated from other locations and that Azores and Madeira groups are highly differentiated from all other groups. This pattern resembles the results observed in the PCoA.

**Table 1:** PERMANOVA pairwise comparisons for β diversity indices based on abundance (Bray–Curtis), presence or absence of microbiome species (Jaccard), sequence distance (Unweighted UniFrac), and sequence distance including abundance (Weighted UniFrac).

Group 1	Group 2	Bray–Curtis	Jaccard	Unweighted UniFrac	Weighted UniFrac
		(*p*-value)	(*p*-value)	(*p*-value)	(*p*-value)
Azores	Canaries	**0.001**	**0.001**	0.113	0.537
	Madeira	**0.001**	**0.001**	**0.001**	**0.004**
	Mainland Portugal	**0.001**	**0.001**	**0.011**	**0.039**
	Selvagens	**0.009**	**0.001**	0.061	0.545
Canaries	Madeira	**0.001**	**0.001**	**0.001**	**0.020**
	Mainland Portugal	**0.001**	**0.002**	0.079	**0.030**
	Selvagens	**0.003**	**0.001**	0.339	0.678
Madeira	Mainland Portugal	**0.002**	**0.001**	**0.001**	**0.001**
	Selvagens	**0.002**	**0.001**	**0.001**	**0.001**
Mainland Portugal	Selvagens	**0.002**	**0.001**	0.177	0.054

Significant *p*-values in bold.

## Discussion

The main results suggest an apparent influence of major currents on the beta diversity of the microbiomes. The genera *Acinetobacter*, *Planctomyces*, *Pseudahrensia* and *Vibrio* dominated overwhelmingly in the studied individuals, especially in Azores and mainland Portugal. The samples from Madeira and Selvagens were dominated by Phyllobacteriacea and *Acinetobacter*, repectively. No consistent trends on microbiome composition were observed along Canarian individuals. These bacteria have been commonly found in coastal molluscs^35–39^ and play a major role in nutrient cycling and influencing host health when in symbiotic relationships^40^. Several *Vibrio* species are stable and frequently associated to marine invertebrates^36–38,41^. *Acinetobacter* species are also present in marine invertebrates as oysters^39^.

The microbiome composition greatly differed among the five studied locations, mainly driven by the differences of the most abundant taxa, i.e. the genera *Acinetobacter*, *Planctomyces*, *Pseudahrensia* and *Vibrio*, and to a lesser extent, by the composition of scarce taxa (< 1% overall abundance). The present results agreed with those reported in previous studies based on molluscs^20,41,42^, and even with works focused on large-scale spatial variability using other marine sessile taxonomic groups as model study, like corals and sponges^43,44^. Neu et al.^20^ showed that composition of microbial communities associated to coastal molluscs greatly differed among species at order and family level. These results are not consistent when comparing to deep corals, where the host is the main driver of microbiome composition rather than the environment^45^.

### Microbiomes and biogeographic patterns

Links between microbiome and biogeography have been previously focused on several organisms, including marine sediment organisms^46^, humans^47^, pigs^48^, gastropods^20^, among others. The microbiome of marine species and marine sediments have revealed that biogeography boundaries and hydrography can play an important role to explain the variation of species among large geographic areas^20,46^.

We observed a strong relationship in alpha diversity of the bacterial microbiome associated with *P. sauciatus* among regions. Moreover, beta-diversity analyses showed three well-differentiated groups, (i) Azores; (ii) Madeira and (iii) mainland Portugal, Selvagens and the Canaries. This grouping is possibly explained by local ecological conditions as occurs with sea anemones^49^, but also by large-scale oceanographic processes, e.g. currents.

Major currents seem to be key drivers to explain the observed variability among locations, with the isolation of two archipelagos, Azores and Madeira, and a group clustering mainland Portugal, Selvagens Islands and the Canaries. The study area is subjected to the North Atlantic Subtropical Gyre (NASG), and specifically it is affected by the Portugal Current and the Canary Current that are the southwestward flow components of the NASG^50^. These currents have been previously shown as driving pathways for connectivity among the species from the Macaronesian archipelagos, and they have been conventionally identified as an ecoregion within the Lusitanian province^51^, also supported by marine phytogeography analysis^52^. However, a different biogeographic classification has been proposed, with 4 ecoregions included in the Lusitanian biogeographic province: (i) the South European Atlantic Shelf, that includes mainland Portugal; (ii) the Saharan upwelling; (iii) the Azores; and (iv) Webbnesia, that includes Madeira, Selvagens and the Canaries^53^. Our results partially agreed with this new classification, but microbiome composition showed a separation of Madeira from Selvagens and the Canaries. These results are in accordance with previous microsatellite studies conducted on the intertidal limpet *Patella candei* complex^54^ that evidenced genetic discontinuity between Madeira and Canarian populations. It needs to be taken into account that genetic analysis and biogeographical patterns reflect contrasting time scales, i.e., centuries for microsatellites and geological time in the case of biogeography.

Migratory events between both archipelagos are very unlikely due to the limited connectivity and genetic differentiation, despite their proximity (*ca.* 400 km) and the presence of Selvagens Islands halfway between Madeira and the Canaries. These genetic patterns are mainly explained by large and meso-scale oceanographic conditions^54^, which origin supposedly dates back to geological time scales. An additional plausible explanation on microbiome content may be found in the seasonal variations of the Canary Current. This major current is originated in the region between Madeira and the African coast, and during winter it tends to be far offshore near to Madeira while in summer occupies a more central position between Madeira and the African coast, affecting in a higher extent the Canary archipelago^55,56^. Hence, oceanographic processes are identified as main drivers of genetic flow in the study region, as it has been concluded by several genetic analysis (microsatellites and microbiome).

Ocean currents constitute pivotal constraints on dispersal and environmental variability of microbiome^57^. This fact has been previously observed in pelagic and benthic sedimentary microbiomes^46,58^, indicating that OTUs are long-distance transported in oceanic masses. In contrast, microbiome co-evolution in isolation occurs minorly in the sampling locations but provides genetic differentiation among them though it does not explain most of the dissimilarity observed in microbiome composition. We herein observed that microbiome composition showed high similarities among populations from distant islands that are also separated by major currents. In the present study, it is suggested that the local environmental may also shape the microbiome of *P. sauciatus.* A combination of both factors, ocean currents and local-scale biogeographic processes may be reliable to explain the observed variability in the sampled Macaronesian archipelagos and mainland Portugal. Further, it needs to be taken into consideration that specimens of *P. sauciatus* may ingest substrate fragments, and acquire rocky-associated microbiome^37^. Our results suggest that the microbiome of *P. sauciatus* is not related to the host population differentiation or the biogeographic pattern of microbiome is more recent that the differentiation recovered with the mtDNA of the host.

### Caveats and study limitations

The samples were collected from one location, with no temporal replication (June–September) where the current connectivity between Madeira and the Canaries is low^56^. Specifically, the sample size (n = 10) may have influenced the whole variability observed among sampling locations, considering that each archipelago comprises different islands, and variations within the same archipelago are expected. Similar studies conducted on intertidal molluscs used similar or lower sample size of each location per species (n = 2–10) from two areas^20^, or even the total number of specimens were lower than the present study (n = 31)^59^. Also, another point to raise is the biased nature of samples where gut and gonad issues were homogenized. Other studies have also combined different organs, revealing its use to accurately describe the biodiversity living within organisms^20,60,61^. Nevertheless, we considered that the use of pooled samples (gut-gonad) provided key information that is of utmost importance as characterization of the microbiome composition of *P. sauciatus*.

PCoA based on unweighted Beta statistics (Jaccard and Unifrac unweighted) revealed a similar trend, where the microbiome of *P. sauciatus* from Selvagens and Madeira revealed a differentiation that could be explained by the major currents. Beta statistics based on presence/absence of species are commonly used to describe a global perspective in large scale areas^62^. The observed discrepancies between PCoA analyses and PERMANOVA could be explained by the higher sensitivity of the analysis to find differences since it looks for pairwise differences overall. Therefore, the differences between PCoA and PERMANOVA results could be mostly explained by different dispersion among groups rather than real differences.

Despite the limitations, the herein information is essential for the study of microbiomes biodiversity and the study of the effects of currents in marine organisms and their microbiomes. Whether major currents can shape the biodiversity patterns of marine organisms, that is certainly a question that needs to be addressed with multiple species.
